# In Memoriam

**Published:** 1902-01

**Authors:** 


### IN MEMORIAM.

Zachary T. Sailer, D.D.S., died June 16, 1901.

Whereas, We have learned of the death of our esteemed fellow-member and Treasurer, Dr. Zachary T. Sailer, who was for many years one of the most earnest and active members of The Alumni Association of the New York College of Dentistry; and

Whereas, We feel that in the demise of Dr. Sailer our Association has sustained a serious loss and a place made vacant that it will be hard to fill; that we have been deprived of an able adviser and stanch friend, a man of integrity and professional worth; therefore be it

*Resolved,* That we do hereby express profound sorrow and regret at the seemingly untimely removal of our respected brother, we will miss his kindly presence, his able advice and loyal service, and his memory will ever be held in tender regard.

*Resolved,* That we extend to the widow and daughter of Dr. Sailer our sympathy in their bereavement, and that these resolutions be spread in full upon the minute-book, and a copy suitably engrossed presented to the family.

John I. Hart,
*President,*
J. Ostram Taylor,
*Secretary,*
Benjamin C. Nash,
*Chairman,*
Benj. F. Luckey,
Chas. A. Du Bois,
*Committee.*

